# Identification of Hub Genes and Potential Molecular Pathogenesis in Substantia Nigra in Parkinson's Disease via Bioinformatics Analysis

**DOI:** 10.1155/2023/6755569

**Published:** 2023-04-14

**Authors:** Yunan Zhou, Zhihui Li, Chunling Chi, Chunmei Li, Meimei Yang, Bin Liu

**Affiliations:** Department of Neurology, The Fourth Affiliated Hospital of Harbin Medical University, Harbin Medical University, Harbin 150001, China

## Abstract

Parkinson's disease (PD) is the second most common neurodegenerative disease, with significant socioeconomic burdens. One of the crucial pathological features of PD is the loss of dopaminergic neurons in the substantia nigra (SN). However, the exact pathogenesis remains unknown. Moreover, therapies to prevent neurodegenerative progress are still being explored. We performed bioinformatics analysis to identify candidate genes and molecular pathogenesis in the SN of patients with PD. We analyzed the expression profiles, GSE49036 and GSE7621, which included 31 SN tissues in PD samples and 17 SN tissues in healthy control samples, and identified 86 common differentially expressed genes (DEGs). Then, GO and KEGG pathway analyses of the identified DEGs were performed to understand the biological processes and significant pathways of PD. Subsequently, a protein-protein interaction network was established, with 15 hub genes and four key modules which were screened in this network. The expression profiles, GSE8397 and GSE42966, were used to verify these hub genes. We demonstrated a decrease in the expression levels of 14 hub genes in the SN tissues of PD samples. Our results indicated that, among the 14 hub genes, DRD2, SLC18A2, and SLC6A3 may participate in the pathogenesis of PD by influencing the function of the dopaminergic synapse. CACNA1E, KCNJ6, and KCNB1 may affect the function of the dopaminergic synapse by regulating ion transmembrane transport. Moreover, we identified eight microRNAs (miRNAs) that can regulate the hub genes and 339 transcription factors (TFs) targeting these hub genes and miRNAs. Subsequently, we established an mTF-miRNA-gene-gTF regulatory network. Together, the identification of DEGs, hub genes, miRNAs, and TFs could provide better insights into the pathogenesis of PD and contribute to the diagnosis and therapies.

## 1. Introduction

Parkinson's disease (PD) is the second most common neurodegenerative disease, with substantial socioeconomic burdens [1]. The prevalence of global PD increased by 155.51% during 1990–2019 [2]. First described by James Parkinson in 1817, PD is characterized by a range of motor symptoms, including bradykinesia, resting tremor, rigidity, and posture instability. In addition, certain non-motor symptoms are also shown to be associated with PD, such as sleep disturbances, autonomic dysfunctions, cognitive and psychiatric dysfunctions, and sensory symptoms [3]. Currently, no available therapies can cause effective prevention of neurodegenerative progress. With the progression of the disease, symptoms that respond poorly to treatment may severely affect the quality of life.

Studies have established that the etiology of PD is multifactorial, which could include genetic factors, environmental factors, nervous system aging, and other factors [4, 5]. The selective loss of dopaminergic neurons in substantia nigra (SN) and the appearance of Lewy bodies in the cytoplasm of the remaining neurons are considered predominant pathological features of PD. In 1893, Bloq and Marinesco proposed the role of SN in the pathological development of PD. In 1912, Friedrich Heinrich Lewy identified the presence of intraneuronal inclusions, now known as Lewy bodies, in the remaining neurons in patients of PD [6]. Studies have shown that the dopaminergic neurons in the ventrolateral tier of the SN are preferentially lost [7]. Moreover, 40–60% of dopaminergic neurons in the nigrostriatal system are lost before the first appearance of the motor symptoms [8]. The loss of dopaminergic neurons in SN could be regulated by several factors, such as oxidative stress, proteasome dysfunction, mitochondrial dysfunction, inflammatory and immune response, apoptosis, and other mechanisms [9, 10]. However, the exact pathogenesis of PD is still unclear. Thus, it is necessary and urgent to examine the pathogenesis of PD and find effective diagnosis and treatment strategies.

In recent years, RNA sequencing and microarray have become indispensable tools for identifying the expression of differential genes, mRNAs, and non-coding RNAs [11]. Furthermore, several datasets of genes expressed in the SN of patients with PD can be downloaded from public databases, such as the Gene Expression Omnibus (GEO). The development of bioinformatics analysis has made it possible to screen, compare, and analyze the existing data and identify the differentially expressed genes (DEGs) that may be related to PD. Systematic studies on the relationship between DEGs could determine the biological processes involved in PD. Therefore, bioinformatics analysis helps explore the biological mechanism and potential biomarkers of PD. The existing bioinformatics research related to PD shows several genes, mRNAs, and non-coding RNAs that may be associated with PD. For example, SLC6A3 is critical in maintaining the integrity of dopaminergic neurons, while SLC18A2 is essential for their survival by contracting intracellular toxicity [12]. ICAM1, also known as CD54, may increase neprilysin levels, essential to treat neurological diseases, including PD [13]. Studies have shown that HRAS may be related to L-DOPA-induced dyskinesia and cognitive impairment [14]. Similarly, miR-338 can decrease mitochondrial activity by reducing the cytochrome c oxidase IV, leading to neuronal damage in SN [15]. These findings using bioinformatics analysis significantly contribute to our understanding of the causes and underlying molecular events of PD. However, additional studies are required to gain a more accurate understanding of PD pathogenesis.

Our study utilized bioinformatics analysis to explore the hub genes and potential molecular mechanisms in the SN of patients with PD, reveal the pathogenesis of PD, find diagnostic markers and therapeutic targets, and provide new perspectives and strategies for the diagnosis, treatment, and new drug development of PD. We identified 86 common DEGs, constructed a protein-protein interaction (PPI) network, and selected 15 hub genes. To understand these genes better, we performed Gene Ontology (GO) and Kyoto Encyclopedia of Genes and Genomes (KEGG) pathway enrichment analyses. Moreover, we identified eight microRNAs (miRNAs) that could regulate the hub genes. By targeting these hub genes and miRNAs, we further identified 339 transcription factors (TFs), thereby establishing an mTF-miRNA-gene-gTF regulatory network. Identifying DEGs, hub genes, miRNAs, and TFs could provide insights into the pathogenesis of PD and contribute to further diagnosis and therapies.

## 2. Materials and Methods

### 2.1. Microarray Data Analysis

The gene expression profiles, GSE49036 and GSE7621, for the SN of patients with PD were obtained from the GEO database (https://www.ncbi.nlm.nih.gov/geo/), an international public repository. Microarray data of GSE49036 and GSE7621 were based on the GPL570 Platform (Affymetrix Human Genome U133 Plus 2.0 Array); GSE49036 included 15 SN tissues of PD samples and eight SN tissues of normal samples, while GSE7621 contained 16 SN tissues of PD samples and 9 SN tissues of normal samples. Additionally, datasets GSE8397 and GSE42966 were obtained from the GEO database to verify hub genes selected from GSE49036 and GSE7621. Microarray data for GSE8397 were based on the GPL96 Platform (Affymetrix Human Genome U133A Array) and GPL97 Platform (Affymetrix Human Genome U133B Array). A and B GeneChip of GSE8397 together contained 24 SN tissues of PD samples and 15 SN tissues of normal samples. Finally, the microarray data for GSE42966 were based on the GPL4133 Platform (Agilent-014850 Whole Human Genome Microarray 4x44K G4112F) and included 9 SN tissues of PD samples and 6 SN tissues of normal samples (Table 1).

### 2.2. Identification of DEGs

DEGs with *P* value <0.05 and |log FC|  ≥  1.0 were selected using GEO2R (https://www.ncbi.nlm.nih.gov/geo/geo2r/). A *P* value <0.05 and log FC ≥ 1.0 indicated upregulated genes, while a *P* value <0.05 and log FC ≤ −1.0 indicated downregulated genes. The common DEGs between GSE49036 and GSE7621 were obtained using Venn online website (http://bioinformatics.psb.ugent.be/webtools/Venn/). GraphPad Prism 9 software was used to make volcano plots to visualize the DEGs better.

### 2.3. GO and KEGG Pathway Analyses of DEGs

GO analysis is one of the most valuable methods for describing the features of genes comprehensively, which includes biological processes (BPs), molecular functions (MFs), and cellular components (CCs). Similarly, the KEGG database explores the functions and biological pathways of genes. GO and KEGG pathway analyses of the overlapping DEGs between GSE49036 and GSE7621 were performed using an online tool called DAVID (https://david.ncifcrf.gov/). Finally, the ggplot2 package in R Studio was applied to depict the bubble plots.

### 2.4. PPI Network Construction and Hub Gene Selection

The Search Tool for the Retrieval of Interacting Genes (STRING (https://string-db.org/)), together with Cytoscape software, was used to build a PPI network (medium confidence: 0.4). MCODE, a plugin in Cytoscape, determined the significant clusters, while the plugin CytoHubba was used to screen the hub genes in the PPI network. GO and KEGG pathway analyses of hub genes and genes in clusters were also predicted by DAVID.

### 2.5. Prediction of Target miRNAs

Two online miRNA databases, TargetScan (https://www.targetscan.org/vert_80/) and miRDB (http://www.mirdb.org/), were used to predict the miRNA targeting hub genes. The intersection of miRNAs obtained above and the differentially expressed miRNAs in SN between PD and normal individuals were obtained using the Venn online website. These miRNAs were considered target miRNAs in the study.

### 2.6. mTF-miRNA-Gene-gTF Regulatory Network Construction

To further explore the functions of the above-found hub genes and target miRNAs in PD pathogenesis, TFs related to hub genes (gTF) and TFs related to target miRNAs (mTF) were predicted by the online database RNAInter (http://www.rnainter.org/). Finally, an mTF-miRNA-gene-gTF regulatory network was established by Cytoscape software.

## 3. Results

### 3.1. Identification of DEGs

**Figure 1:**
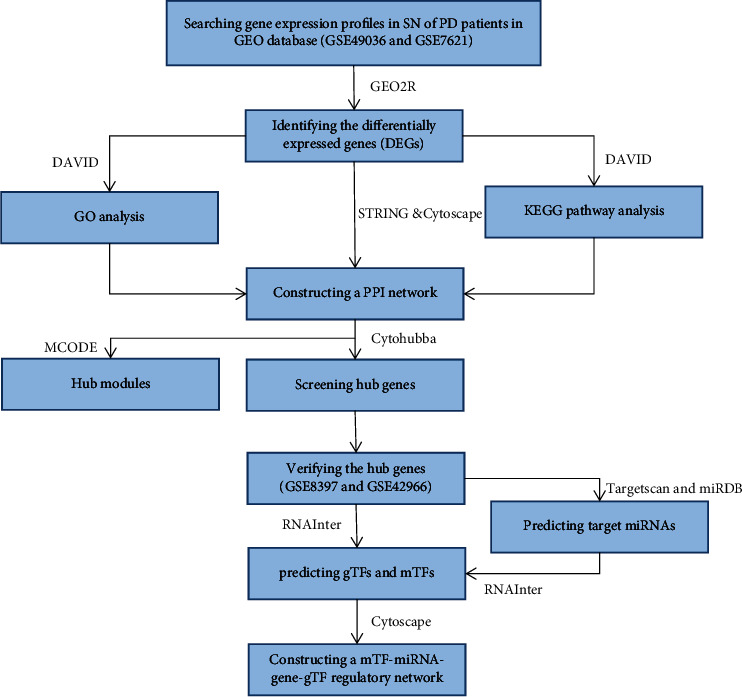
The concise diagram of workflow.

**Figure 2:**
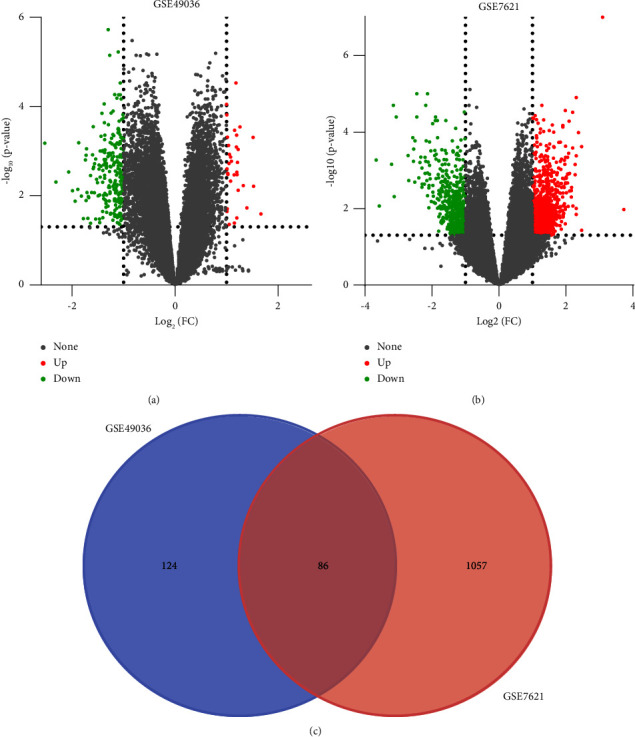
The volcano plots of differential expression of data between two sets of samples, Venn diagram. (a) The GSE49036 dataset. (b) The GSE7621 dataset. The red points represent upregulated genes screened based on fold change ≥ 1.0 and *P* value <0.05. The green points represent the downregulated genes screened based on fold change ≤−1.0 and *P* value <0.05. The black points represent genes with no significant change. (c) Venn diagram of DEGs between GSE49036 and GSE7621. The two datasets show an overlap of 86 genes. DEGs, differentially expressed genes.

The concise diagram of workflow is summarized in Figure 1. We selected two datasets, GSE49036 (15 SN tissues of PD samples and eight SN tissues of normal samples) and GSE7621 (16 SN tissues of PD samples and nine SN tissues of normal samples), for our study. Based on the criteria of *P* value <0.05 and |log FC|  ≥  1.0, we obtained 253 DEGs (29 upregulated genes and 224 downregulated genes) from GSE49036 and 1236 DEGs (732 upregulated genes and 504 downregulated genes) from GSE7621. We used volcano plots to visualize the DEGs in GSE49036 and GSE7621 (Figures 2(a) and 2(b)). Subsequently, we identified 86 overlapping DEGs (2 upregulated genes and 84 downregulated genes) between GSE49036 and GSE7621 (Figure 2(c), Supplementary Table 1).

### 3.2. GO and KEGG Pathway Analyses of DEGs

**Figure 3:**
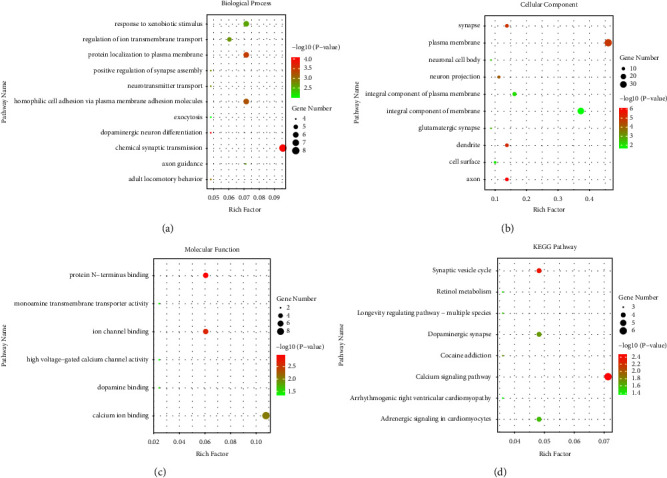
Functional enrichment of common DEGs between GSE49036 and GSE7621. (a) BP, biological process. (b) CC, cellular component. (c) MF, molecular function. (d) KEGG pathway enrichment analysis. DEGs, differentially expressed genes.

For a comprehensive understanding of the DEGs, we performed GO and KEGG pathway analyses (*P* value <0.05) using DAVID. The results of the GO analysis are presented in Table 2 and Figures 3(a)–3(c). For BP, these common DEGs were significantly enriched in chemical synaptic transmission, protein localization to the plasma membrane, homophilic cell adhesion via plasma membrane adhesion molecules, response to xenobiotic stimulus, axon guidance, regulation of ion transmembrane transport, dopaminergic neuron differentiation, adult locomotory behavior, neurotransmitter transport, positive regulation of synapse assembly, and exocytosis. For CC, the common DEGs were mainly enriched in integral components of the plasma membrane, axon, dendrite, synapse, neuron projection, cell surface, glutamatergic synapse, and neuronal cell body. For MF, the common DEGs were enriched in calcium ion binding, protein N-terminus binding, ion channel binding, dopamine binding, monoamine transmembrane transporter activity, and high voltage-gated calcium channel activity. KEGG pathways were mainly enriched in the calcium signaling pathway, dopaminergic synapse, synaptic vesicle cycle, adrenergic signaling in cardiomyocytes, cocaine addiction, longevity regulating pathway-multiple species, retinol metabolism, and arrhythmogenic right ventricular cardiomyopathy (Table 3 and Figure 3(d)).

### 3.3. PPI Network Construction and Hub Gene Identification

**Figure 4:**
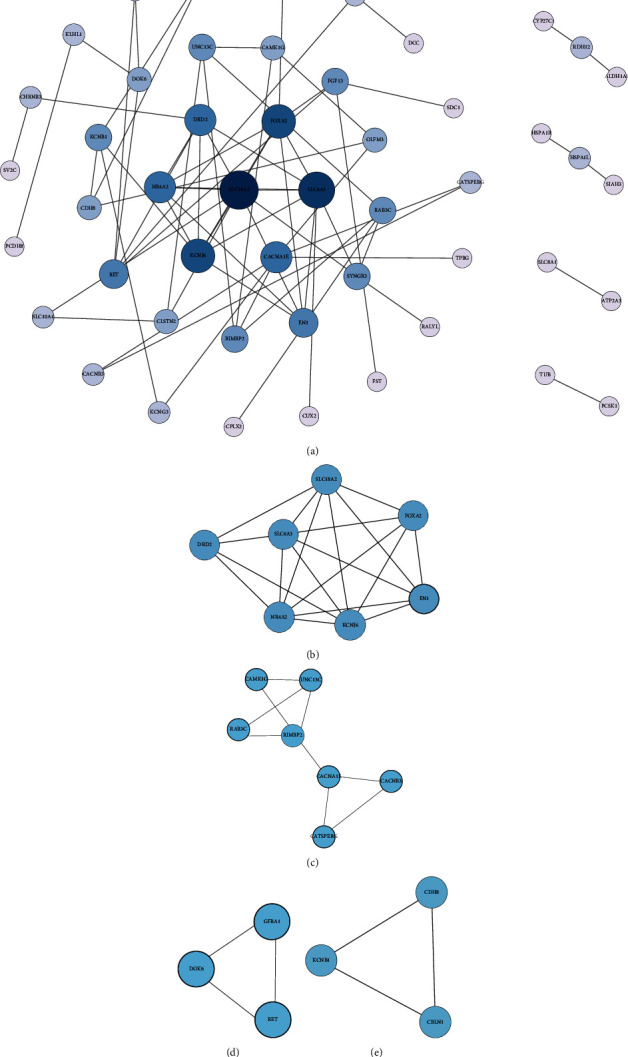
PPI network and the most significant module of DEGs. (a) The PPI network of DEGs was constructed using Cytoscape with 49 nodes and 69 edges. The size and color of the circle indicate the node degree. (b–e) The four most significant modules obtained from the PPI network. DEGs, differentially expressed genes; PPI, protein-protein interaction.

Using the data obtained above, we constructed a PPI network consisting of 49 nodes and 69 edges (Figure 4(a)). The MCODE plugin in Cytoscape generated four modules (Figures 4(b)–4(e)); cluster 1, comprising 7 nodes and 19 edges, got the highest score (score: 6.333), while cluster 2 (7 nodes and 9 edges), cluster 3 (3 nodes and 3 edges) and cluster 4 (3 nodes and 3 edges) had the same score (score: 3). The results of GO and KEGG analyses for these four modules suggest that DEGs are enriched mainly in transmembrane transport, chemical synaptic transmission, the biological process of locomotory behavior, and PD (*P* value <0.05; Supplementary Tables 2 and 3). However, we did not find any hits in the MF analysis for cluster 4 and KEGG pathway analysis for clusters 2, 3, and 4.

**Figure 5:**
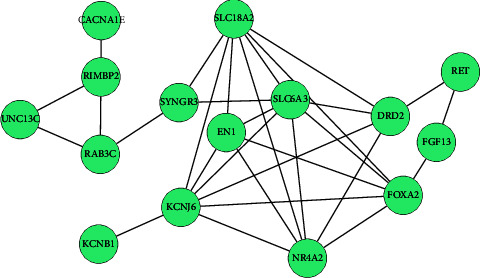
Interaction network of 15 hub genes. The green color represents the downregulated genes screened based on fold change ≤ −1.0 and *P* value <0.05.

Then, the plugin CytoHubba provided us top 15 most significant genes (SLC18A2, SLC6A3, KCNJ6, FOXA2, NR4A2, CACNA1E, DRD2, RET, EN1, FGF13, SYNGR3, RIMBP2, UNC13C, KCNB1, and RAB3C), which were considered as hub genes. The degrees of 15 hub genes are all greater than or equal to 4, and SLC18A2 got the highest degree of 9 (Table 4 and Figure 5). Moreover, all 15 hub genes obtained were downregulated genes. To further explore these genes, we conducted GO and KEGG pathway analyses (Table 5). For BP, these hub genes were significantly enriched in dopaminergic neuron differentiation, locomotory behavior, regulation of ion transmembrane transport, and chemical synaptic transmission. For CC, hub genes were enriched in the plasma membrane, axon, dendrite, synapse, and neuronal cell body. For MF, hub genes were enriched in protein N-terminus binding, dopamine binding, and monoamine transmembrane transporter activity. On the other hand, for the KEGG pathway, hub genes were enriched in the dopaminergic synapse, PD, cocaine addiction, synaptic vesicle cycle, and alcoholism.

### 3.4. Validation of the Hub Genes

**Figure 6:**
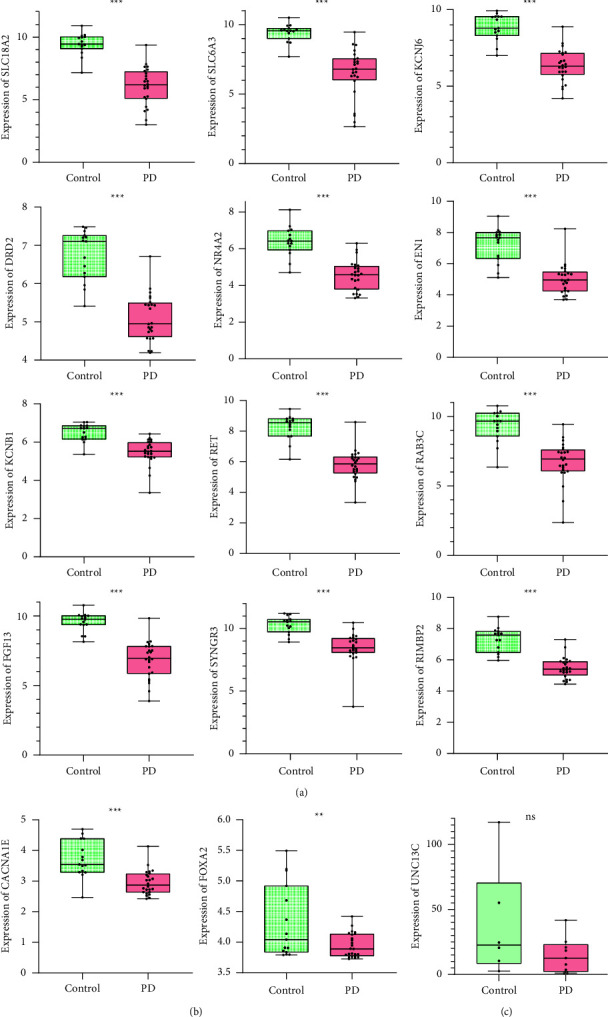
The box plots of the expressions of 15 hub genes in SN of patients with PD and control samples. ^*∗∗∗*^The expression levels of hub genes in SN tissues of PD samples were significantly decreased compared with those in control samples (*P* value <0.01 and log FC ≤ −1.0). ^*∗∗*^The gene expression level was reduced in SN tissues of PD samples, but not significantly (*P* value <0.05 and −1 < log FC < 0). ns: the gene expression level was decreased in SN tissues of PD samples, but it was not statistically significant (*P* value >0.05).

To reinforce the reliability of hub genes in our study, we verified the 15 hub genes in GSE8397 and GSE42966 and performed the box plots using GraphPad Prism 9 software (Figure 6). The expression levels of the 12 hub genes (SLC18A2, SLC6A3, KCNJ6, NR4A2, DRD2, RET, EN1, FGF13, SYNGR3, RIMBP2, KCNB1, and RAB3C) in SN tissues of PD samples were significantly decreased compared with those of normal samples (*P* value <0.01 and log FC ≤ −1.0). While the expression levels of FOXA2 and CACNA1E were also reduced in SN tissues of PD samples, they were not statistically significant (*P* value < 0.05 but −1< log FC < 0). On the other hand, the levels of UNC13C did not show any difference (*P* value >0.05). Together, our results indicated that the expression levels of 14 hub genes (SLC18A2, SLC6A3, KCNJ6, FOXA2, NR4A2, CACNA1E, DRD2, RET, EN1, FGF13, SYNGR3, RIMBP2, KCNB1, and RAB3C) were decreased in SN tissues of PD samples.

### 3.5. Prediction of Target miRNAs

We uploaded the 14 hub genes validated above onto the miRNA databases TargetScan and miRDB, respectively. From this, we obtained 1486 intersections using the Venn online website. Next, we selected 43 miRNAs from previous studies in PubMed that have been verified to express differentially in SN tissues between PD and healthy control samples. Further, using the Venn online website, we uploaded 1486 miRNAs from TargetScan and miRDB and 43 miRNAs from PubMed. Finally, we acquired eight target miRNAs (hsa-miR-532-5p, hsa-miR-23b-3p, hsa-miR-198, hsa-miR-330-5p, hsa-miR-339-5p, hsa-miR-485-5p, hsa-miR-34a-5p, and hsa-miR-7-5p). Details of these eight target miRNAs are shown in Table 6.

### 3.6. Prediction of Target TFs and Construction of mTF-miRNA-Gene-gTF Regulatory Network

**Figure 7:**
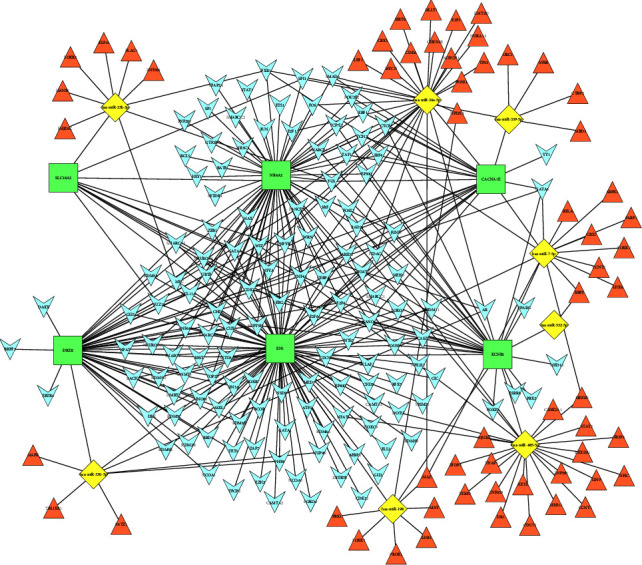
The mTF-miRNA-gene-gTF regulatory network. The square-shaped green nodes represent hub genes, the diamond-shaped yellow nodes represent miRNAs, the V-shaped blue nodes represent the TFs associated with hub genes (gTFs), and the triangle-shaped orange nodes represent the TFs associated with miRNAs (mTFs).

To better understand the 14 hub genes and eight target miRNAs found above, TFs targeting hub genes (gTF) and TFs targeting miRNAs (mTF) were identified by the online database RNAInter. Moreover, we constructed an mTF-miRNA-gene-gTF regulatory network using the software Cytoscape (Figure 7). This network consisted of 208 nodes and 351 edges, involving six hub genes, eight target miRNAs, and 194 target TFs. The top seven TFs of the network with the highest degrees (degree ≥5) were NHF4A, CDX2, FUS, E2F4, E2F6, ERG, and SUPT5H.

## 4. Discussion

Several efforts have been made to explore the pathogenesis of PD in recent years. However, the pathogenesis of PD is still unclear, and its effective therapy still needs more studies. With the development of bioinformatics, microarray has become an indispensable tool to identify the expression of differential genes, mRNAs, and non-coding RNAs. Moreover, several datasets of genes expressed in SN of PD have also been uploaded to the GEO database. In the past years, most studies only analyzed one microarray dataset, leading to incomprehensive results.

This study explored the potential pathogenesis in SN of PD via bioinformatics analysis with different microarray datasets. We identified 86 DEGs that were significantly enriched in various metabolism pathways. Further, we found that 14 hub genes, eight miRNAs, and seven TFs may play important roles in the pathogenesis of PD. GO and KEGG pathway analyses of hub genes suggest that the regulation of dopaminergic synaptic transmission might be involved in the pathogenesis of PD.

We found that three hub genes, DRD2, SLC18A2, and SLC6A3, in the PPI network were significantly enriched in the dopaminergic synapse, whereas SLC18A2 and SLC6A3 exhibited the highest degree of 9 and 8, respectively. DRD2 encodes the D2 subtype of the dopamine receptor. Recent studies show that dopamine agonists with high selectivity for DRD2 have already been used to improve symptoms in patients with PD [16]. DRD2 is related to peak-dose dyskinesias induced by levodopa in patients with PD [17]. The DRD2 polymorphism, rs1076560 DRD2 G > T, might influence gait function for patients with PD [18]. A clinical trial with 217 patients with PD on levodo pa therapy showed that DRD2 rs1799732 is an independent predictor of gastrointestinal symptoms associated with levodopa therapy [19]. SLC6A3 and SLC18A2 are the other two crucial candidate genes in sporadic PD. Encoded by SLC6A3, dopamine transporter (DAT) is the protein with the most selective expression of the most damaged dopaminergic neurons in patients with PD. Na + -K-ATPases on the plasma membrane can generate ion gradients. DAT reuptakes dopamine into presynaptic neurons from the synaptic cleft depending on the cotransport of Na+ and Cl− down the ions' concentration gradients [20]. Mainly present on the neuron terminals in SN, DAT is necessary for dopaminergic neurotransmission to control its intensity and duration [21]. A randomized trial by Moreau et al. showed that methylphenidate, an inhibitor of SLC6A3, can reduce the severity of gait hypokinesia and freezing in patients with advanced PD who received subthalamic nucleus stimulation [22]. SLC18A2 encodes vesicular monoamine transporter 2 (VMAT2), which can transport cytoplasmic monoamines into synaptic vesicles for storage, and then releases them extracellularly in the central nervous system, driven by H+ electrochemical force. Therefore, the concentrations of monoamine neurotransmitters in synaptic vesicles can maintain a high level, and those in the cytoplasm can maintain a low level. On the contrary, the decrease of VMAT2 leads to the increase of monoamine neurotransmitters in cytoplasm, which further leads to the formation of cytotoxic free radicals and finally results in the degeneration of neurons [23]. Taylor et al. created a VMAT2-deficient mouse model of PD and demonstrated progressive motor and non-motor symptoms and neurodegeneration in SN, locus coeruleus, and dorsal raphe [24]. Pifl et al. performed autopsies on six patients with PD and four healthy controls and gained dopamine storage vesicles from their striatum. They found that in patients with PD, the level of VMAT2 and synaptic vesicular dopamine uptake was significantly reduced, and dopamine storage impairment was located in the VMAT2 itself [25]. Interestingly, a decrease in vesicular function because of SLC18A2 mutation could lead to brain dopamine-serotonin vesicular transport disease, including infantile parkinsonism-dystonia-2. Several patients with brain dopamine-serotonin vesicular transport disease have been found in the world. The homozygous c.710C > T (p.Pro237His) transition in SLC18A2 has been identified in a 6-month-old male infant of China, two New Zealand siblings of European descent, and a 7-year-old female of Iraq [26–28]. Moreover, another variant, c.1160C⟶T in SLC18A2, has been observed in eight children in a Saudi Arabian family [29]. These observations are consistent with our results that the expression of DRD2, SLC18A2, and SLC6A3 in patients of PD is significantly reduced. So, we speculated that DRD2, SLC18A2, and SLC6A3 might participate in the pathogenesis of PD by influencing the function of the dopaminergic synapse.

GO analysis showed that CACNA1E, KCNJ6, and KCNB1 participated in the regulation of ion transmembrane transport. Ion channels are proteins that generate and modulate electricity across biological membranes. CACNA1E encodes the high-voltage-activated Cav2.3 type R calcium channel. Voltage-gated Ca2+ channels, whose primary function is to initiate the synaptic transmission and neurotransmitter release, consist of 5 distinct subunits (*α*1, *α*2, *β*, *γ*, and *δ*). The *α*1 subunit can be divided into 3 subfamilies, namely, Cav1, Cav2, and Cav3 [30]. It has been reported that among all voltage-gated Ca2+ channel subtypes in adult SN dopaminergic neurons, Cav2.3 accounts for the most significant proportion. In SN dopaminergic neurons, the activity that generates oscillatory increases in free cytosolic Ca2+ levels, which are thought to impart mitochondrial stress and render these neurons more vulnerable to degeneration by PD stressors. In Cav2.3 knockout mice and Cav2.3 inhibitor SNX-482 using mice, the activity-associated nigral somatic Ca2+ signals reduced, which indicates that Cav2.3 contributes to neurodegeneration [31]. KCNJ6 encodes a potassium channel subunit called GIRK2, which belongs to the G-protein-gated inwardly rectifying potassium channel (GIRK) family. GIRK can be activated by ligand-stimulated G protein-coupled receptors (GPCRs), such as dopaminergic D2 receptors. Thus, the permeability of GIRK to K+ increases while the excitability of neurons decreases [32, 33]. KCNB1 encodes an ion channel called the delayed rectifier voltage-gated K+ channel KCNB1 (Kv2.1). Studies using the traumatic brain in mouse models indicated that the oxidation of Kv2.1 may cause neurodegeneration and cognitive impairment [34]. However, the specific function and mechanism of KCNB1 in PD need further examination. These conclusions are consistent with our GO analysis results that CACNA1E and KCNJ6 are both enriched in the regulation of ion transmembrane transport and dopaminergic synapse, but KCNB1 is only enriched in the regulation of ion transmembrane transport. Therefore, our results suggest that CACNA1E, KCNJ6, and KCNB1 might influence the function of the dopaminergic synapse by participating in the regulation of ion transmembrane transport.

GO analysis in our study also showed that NR4A2, FOXA2, and EN1 are enriched in dopaminergic neuron differentiation and adult locomotory behavior. NR4A2, also called Nurr1, is expressed predominantly in the central nervous system, especially in SN. NR4A2 is related to the differentiation of the dopaminergic neurons in SN via activating the transcription of tyrosine hydroxylase and enhancing the expression of DAT [35–37]. Xu et al. performed heteroduplex analysis and sequencing analysis for the polymorphisms and mutations of NR4A2 in 225 patients with PD and 221 healthy control individuals. They found that a homozygous 7048G7049 polymorphism in intron 6 of the NR4A2 was higher in patients with PD than in healthy people. Their analysis provides evidence for the association between NR4A2 and PD [38]. FOXA2 encodes a member of the forkhead class of DNA-binding proteins, which have been suggested to enhance the expression of the Nurr1-induced DA phenotype [39]. EN1 encodes a homeodomain TF, which is necessary for the development of neurons in the midbrain, cerebellum, hindbrain, and spinal cord [40]. Simon et al. investigated the homeodomain TFs En-1 and En-2 in mice and found an increased En-1 in almost all dopaminergic neurons in SN and ventral tegmentum, but it was not required for their specification [41]. These studies further support the current findings.

Additionally, our analysis showed that SYNGR3, RET, FGF13, RIMBP2, and RAB3C were downregulated in the SN of patients with PD and were the hub genes in the PPI network. SYNGR3 encodes an integral membrane protein called synaptogyrin-3, located on the synaptic vesicular membrane and involved in synaptic vesicular trafficking. The impairment of synaptic vesicular trafficking is one of the earliest pathological processes involved in PD [42]. In mice, synaptogyrin-3 interacts with DAT and increases its activity. This effect could be abolished in the presence of reserpine, a VMAT2 inhibitor [43]. Similar to our findings, Simunovic et al. analyzed the gene expression profiling of SN pars compacta in patients with PD and control individuals and found a reduction in the expression of SYNGR3 [44]. RET is a member of the cadherin superfamily. Glial cell line-derived neurotrophic factor (GDNF), which is one of the ligands of RET, can bind with the glycosylphosphatidylinositol- (GPI-) linked GDNF family receptor alpha 1 (GFR*α*1). The combination of GDNF and GFR*α*1 can bind with RET to activate its intracellular tyrosine kinase activity [45]. Activated RET, in turn, activates the intracellular mitogen-activated protein kinase (MAPK), Akt (protein kinase B), and Src signaling cascades which can maintain the survival and regeneration of DA neurons [46]. Thus, GDNF is suggested to be one of the target-derived neurotrophic factors for the development of DA neurons and one of the factors that can maintain the survival of midbrain DA neurons [47, 48]. Experiments in mice models confirmed that RET was crucial to the nigrostriatal DA system preservation. RET ablations in mice could lead to progressive loss of DA neurons and degeneration of DA nerve terminals in the striatum [49]. FGF13 encodes a protein that belongs to the fibroblast growth factor (FGF) family. Anatomic studies and electrophysiological recordings showed that FGF13 plays a powerful role in regulating excitability for hippocampal neurons [50]. RIMBP stands for Rab-interacting molecule- (RIM-) binding protein, whose interactions with Cav have been confirmed [51]. RIM-BP2 might have the most robust association with synaptic transmission [52]. RAB3C encodes a small GTPase. Mollard et al. concluded that similar to RAB3A, RAB3C is also localized on synaptic vesicles and is involved in vesicle trafficking in the nervous system [53]. However, there is no relevant report about the functions of FGF13, RIMBP2, and RAB3C in PD; therefore, further research is needed to understand their contribution to PD.

miRNAs are small RNA molecules that can regulate gene expressions after transcription [54]. In our study, eight target miRNAs (hsa-miR-532-5p, hsa-miR-23b-3p, hsa-miR-198, hsa-miR-330-5p, hsa-miR-339-5p, hsa-miR-485-5p, hsa-miR-34a-5p, and hsa-miR-7-5p) were found to be associated with SN in individuals with PD. hsa-miR-532-5p has been shown to be associated with KCNB1. Briggs et al. studied SN in eight idiopathic patients with PD obtained from the Harvard Brain Tissue Resource Center and found a downregulation in hsa-miR-532-5p [55]. A meta-analysis showed that the miRNAs hsa-miR-23b-3p, hsa-miR-7-5p, and hsa-miR-34a-5p were associated with regulating the expression of SNCA [56]. On the other hand, the expression of hsa-miR-198, hsa-miR-330-5p, hsa-miR-339-5p, and hsa-miR-485-5p was significantly different between SN tissues in eight PD patients and four controls [57]. Consistently, these eight miRNAs were found to be associated with six hub genes (SLC18A2, NR4A2, CACNA1E, DRD2, EN1, and KCNB1) in our study. KCNB1 and CACNA1E are considered the most significant genes among these genes above and can be regulated by four miRNAs. Therefore, eight miRNAs might be associated with the pathogenesis of PD. However, future studies should pay more attention to these miRNAs.

Finally, we constructed an mTF-miRNA-gene-gTF regulatory network. We obtained 248 gTFs and 91 mTFs, which can regulate these six hub genes and eight miRNAs. EN1 and NR4A2 were found to be regulated by the highest number of TFs (90 and 61 TFs, respectively). Moreover, TFs, such as HNF4A, FUS, CDX2, SUPT5X, ERG, E2F4, and E2F6, could regulate the most significant number of genes and miRNAs in this network. HNF4A, hepatocyte nuclear factor 4 alpha, might influence gluconeogenesis, diabetes, and lipid homeostasis [58, 59]. Previous studies have highlighted the interaction between HNF4A and peroxisome proliferator activator receptor gamma (PPAR-*γ*), a potential therapeutic target in PD [60]. Further, a meta-analysis has identified HNF4A as the most significantly upregulated TF in the blood of patients with PD, while its relative abundance correlated with disease severity in patients with PD [61]. Further studies are required to examine the correlation of FUS, CDX2, SUPT5X, ERG, E2F4, and E2F6 TFs with PD.

## 5. Conclusions

We performed bioinformatics analysis on microarray datasets from studies on SN of patients with PD. Our study identified 86 common DEGs and 14 hub genes in the PPI network. Among them, DRD2, SLC18A2, and SLC6A3 were shown to participate in the pathogenesis of PD by influencing the function of the dopaminergic synapse. CACNA1E, KCNJ6, and KCNB1 might affect the function of the dopaminergic synapse by regulating ion transmembrane transport. Further, we predicted eight miRNAs (hsa-miR-532-5p, hsa-miR-198, hsa-miR-23b-3p, hsa-miR-339-5p, hsa-miR-330-5p, hsa-miR-485-5p, hsa-miR-34a-5p, and hsa-miR-7-5p) which were confirmed to be related to the SN of patients with PD. Using the obtained 248 gTFs and 91 mTFs, we further constructed an mTF-miRNA-gene-gTF regulatory network. Finally, TFs, such as HNF4A, FUS, CDX2, SUPT5X, ERG, E2F4, and E2F6, were found to regulate most number of genes and miRNAs. However, due to the lack of SN samples, additional experiments could not be conducted, and therefore, further studies are required to provide deeper insights into the pathogenesis of PD.

## Figures and Tables

**Table 1:** The fundamental information of microarray datasets from GEO.

GEO accession	Platform	Samples		Authors	Publication	Year
		PD	Control			
GSE49036	GPL570	15	8	Dijkstra et al.	PLoS One	2015
GSE7621	GPL570	16	9	Ffrench-Mullen et al.	PLoS Genet	2007
GSE8397	GPL96	24	15	Moran et al.	Neurogenetics	2006
	GPL97	24	15	Moran et al.	Neurogenetics	2007
GSE42966	GPL4133	9	6	Bando et al.	—	—

**Table 2:** Ontological analysis of differently expressed genes in GSE49036 and GSE7621.

Category	GO ID	Term	*P* value	Count	Genes
Biological Processes	0007268	Chemical synaptic transmission	9.70*E* − 05	8	CACNB3/CBLN1/CACNA1E/SV2C/CHRNB3/PCDH8/SLC18A2/UNC13C
	0072659	Protein localization to the plasma membrane	3.80*E* − 04	6	RAB3C/ANK1/CACNB3/FGF13/KCNB1/TPBG
	0007156	Homophilic cell adhesion via plasma membrane adhesion molecules	5.90*E* − 04	6	CDH8/CLSTN2/CNTN6/PCDH8/RET/ROBO2
	0009410	Response to xenobiotic stimulus	2.60*E* − 03	6	SHANK2/DRD2/PCSK1/SLC8A1/SLC6A3/RET
	0034765	Regulation of ion transmembrane transport	2.00*E* − 03	5	KCNB1/CACNB3/CACNA1E/KCNJ6/KCNG3
	0007411	Axon guidance	2.50*E* − 03	4	DCC/CNTN6/ RET/ROBO2
	0071542	Dopaminergic neuron differentiation	8.80*E* − 05	4	RSPO2/EN1/FOXA2/NR4A2
	0008344	Adult locomotory behavior	9.80*E* − 04	4	SHANK2/EN1/FOXA2/NR4A2
	0006836	Neurotransmitter transport	1.80*E* − 03	4	CPLX2/SLC6A3/SLC18A2/SV2C
	0051965	Positive regulation of synapse assembly	1.80*E* − 03	4	CLSTN2/CBLN1/CUX2/TPBG
	0006887	Exocytosis	9.90*E* − 03	4	ANK1/UNC13C/CPLX2/KCNB1

Cellular Components	0005886	Plasma membrane	1.50*E* − 05	37	DCC/DIRAS2/TUB/GPR26/GPRC5A/GFRA1/RAB27B/RAB3C/RIMBP2/SHANK2/AGTR1/ANK1/CDH8/DRD2/CACNA1E/CNTN6/CAMK1G/CHRNB3/CATSPERG/FGF13/KCNJ6/KCNG3/RET/KCNB1/PCDH8/ROBO2/SLC10A4/STYK1/SLC18A2/SLC2A13/SLC6A3/SLC8A1/SSTR1/SV2C/SDC1/TPBG/UNC13C
	0016021	Integral components of membrane	2.00*E* − 02	30	ATP2A3/DCC/RET/CDH8/CHRNB3/GPR161/GPR26/GPRC5A/SHANK2/AGTR1/B4GALT6/CNTN6/DAPL1/DLK1/DRD2/SV2C/KCNG3/KCNB1/PCDH8/REEP1/ROBO2/SLC10A4/STYK1/SLC2A13/SLC35D3/SLC6A3/SLC8A1/SUSD1/SYNGR3/SDC1
	0005887	Integral components of plasma membrane	7.70*E* − 03	13	GPRC5A/AGTR1/CHRNB3/PCDH8/DRD2/SLC18A2/RET/SLC2A13/SDC1/SLC6A3/SLC8A1/SSTR1/TPBG
	0030424	Axon	7.90*E* − 07	11	DCC/ALDH1A1/GFRA1/DRD2/RET/CNTN6/SLC6A3/FGF13/KCNB1/ROBO2/SLC8A1
	0030425	Dendrite	8.10*E* − 06	11	CLSTN2/CPLX2/RET/DRD2/FGF13/SLC8A1/KLHL1/KCNB1/PCSK1/PCDH8/TPBG
	0045202	Synapse	1.20*E* − 05	11	RIMBP2/ALDH1A1/CACNB3/CBLN1/CACNA1E/CPLX2/CHRNB3/DRD2/OLFM3/SLC8A1/UNC13C
	0043005	Neuron projection	6.80*E* − 05	9	SHANK2/PCSK1/CAMK1G/ANK1/CHRNB3/FGF13/SLC6A3/SSTR1/SV2C
	0009986	Cell surface	9.90*E* − 03	8	DCC/CLSTN2/TPBG/RSPO2/KCNB1/ROBO2/SDC1/SLC6A3
	0098978	Glutamatergic synapse	2.70*E* − 03	7	SHANK2/CDH8/CLSTN2/CBLN1/CPLX2/PCDH8/DRD2
	0043025	Neuronal cell body	3.90*E* − 03	7	GFRA1/SHANK2/CACNA1E/KLHL1/RET/SLC6A3/SLC8A1

Molecular Functions	0005509	Calcium ion binding	8.80*E* − 03	9	CDH8/CACNA1E/CLSTN2/DLK1/RET/PCDH8/SLC8A1/SUSD1/UNC13C
	0047485	Protein N-terminus binding	1.10*E* − 03	5	HSPA1B/KLHL13/KCNB1/SLC6A3/SYNGR3
	0044325	Ion channel binding	2.20*E* − 03	5	ATP2A3/SLC8A1/ANK1/FGF13/KCNB1
	0035240	Dopamine binding	2.70*E* − 02	2	SLC6A3/DRD2
	0008504	Monoamine transmembrane transporter activity	3.50*E* − 02	2	SLC6A3/SLC8A2
	0008331	High voltage-gated calcium channel activity	4.30*E* − 02	2	CACNB3/CACNA1E

**Table 3:** KEGG pathway analysis of differentially expressed genes in GSE49036 and GSE7621.

Category	Term	*P* value	Count	Genes
KEGG pathways	Calcium signaling pathway	3.40*E* − 03	6	ATP2A3/AGTR1/CACNA1E/RET/CAMK1G/SLC8A1
	Synaptic vesicle cycle	4.50*E* − 03	4	CPLX2/SLC18A2/SLC6A3/UNC13C
	Dopaminergic synapse	1.90*E* − 02	4	DRD2/KCNJ6/SLC18A2/SLC6A3
	Adrenergic signaling in cardiomyocytes	2.60*E* − 02	4	ATP2A3/AGTR1/CACNB3/SLC8A1
	Cocaine addiction	1.90*E* − 02	3	DRD2/SLC18A2/SLC6A3
	Longevity regulating pathway-multiple species	2.90*E* − 02	3	FOXA2/HSPA1L/HSPA1B
	Retinol metabolism	3.40*E* − 02	3	ALDH1A1/RDH12/CYP27C1
	Arrhythmogenic right ventricular cardiomyopathy	4.30*E* − 02	3	ATP2A3/CACNB3/SLC8A1

**Table 4:** The information of the top 15 hub genes.

Gene symbol	Description	Degree	Up/down	Chromosome	Location
SLC18A2	Solute carrier family 18 member A2	9	Down	10	10q25.3
SLC6A3	Solute carrier family 6 member 3	8	Down	5	5p15.33
KCNJ6	Potassium inwardly rectifying channel subfamily J member 6	7	Down	21	21q22.13
FOXA2	Forkhead box A2	7	Down	20	20p11.21
NR4A2	Nuclear receptor subfamily 4 group A member 2	6	Down	2	2q24.1
CACNA1E	Calcium voltage-gated channel subunit alpha1 E	6	Down	1	1q25.3
DRD2	Dopamine receptor D2	6	Down	11	11q23.2
RET	Ret proto-oncogene	5	Down	10	10q11.21
EN1	Engrailed homeobox 1	5	Down	2	2q14.2
FGF13	Fibroblast growth factor 13	4	Down	X	Xq26.3-q27.1
SYNGR3	Synaptogyrin-3	4	Down	16	16p13.3
RIMBP2	RIMS binding protein 2	4	Down	12	12q24.33
UNC13C	Unc-13 homolog C	4	Down	15	15q21.3
KCNB1	Potassium voltage-gated channel subfamily B member 1	4	Down	20	20q13.13
RAB3C	RAB3C, member RAS oncogene family	4	Down	5	5q11.2

**Table 5:** The ontological analysis of hub genes.

Category	GO ID	Term	*P* value	Count	Genes
Biological processes	0071542	Dopaminergic neuron differentiation	1.10*E* − 04	3	EN1/FOXA2/NR4A2
	0042220	Response to cocaine	3.40*E* − 04	3	DRD2/EN1/SLC6A3
	0001975	Response to amphetamine	3.50*E* − 04	3	DRD2/NR4A2/SLC18A2
	000834	Adult locomotory behavior	5.60*E* − 04	3	EN1/FOXA2/NR4A2
	0007626	Locomotory behavior	1.70*E* − 03	3	DRD2/SLC6A3/SLC18A2
	0034765	Regulation of ion transmembrane transport	4.10*E* − 03	3	CACNA1E/KCNJ6/KCNB1
	0072659	Protein localization to the plasma membrane	5.40*E* − 03	3	RAB3C/FGF13/KCNB1
	0009410	Response to xenobiotic stimulus	1.20*E* − 02	3	DRD2/RET/SLC6A3
	0007268	Chemical synaptic transmission	1.60*E* − 02	3	CACNA1E/UNC13C/SLC18A2
	0009410	Response to drug	1.80*E* − 02	3	DRD2/RET/SLC6A3

Cellular components	0005886	Plasma membrane	2.90*E* − 04	11	RAB3C/DRD2/RET/RIMBP2/UNC13C/FGF13/KCNJ6/CACNA1E/KCNB1/SLC6A3/SLC18A2
	0030424	Axon	7.60*E* − 05	5	DRD2/FGF13/RET/KCNB1/SLC6A3
	0030672	Synaptic vesicle membrane	2.00*E* − 05	4	DRD2/SYNGR3/SLC18A2/UNC13C
	0030425	Dendrite	3.50*E* − 03	4	DRD2/FGF13/RET/KCNB1
	0045202	Synapse	4.20*E* − 03	4	DRD2/RIMBP2/UNC13C/CACNA1E
	0016328	Lateral plasma membrane	1.20*E* − 03	3	DRD2/FGF13/KCNB1
	0008021	Synaptic vesicle	3.80*E* − 03	3	RAB3C/SLC18A2/SYNGR3
	0043025	Neuronal cell body	3.00*E* − 02	3	RET/SLC6A3/CACNA1E

Molecular functions	0047485	Protein N-terminus binding	2.80*E* − 03	3	KCNB1/SLC6A3/SYNGR3
	0035240	Dopamine binding	4.80*E* − 03	2	DRD2/SLC6A3
	0008504	Monoamine transmembrane transporter activity	6.20*E* − 03	2	SLC18A2/SLC6A3

KEGG pathways		Dopaminergic synapse	2.20*E* − 04	4	DRD2/SLC6A3/KCNJ6/SLC18A2
		Cocaine addiction	9.80*E* − 04	3	DRD2/SLC18A2/SLC6A3
		Synaptic vesicle cycle	2.50*E* − 03	3	SLC18A2/SLC6A3/UNC13C
		Alcoholism	1.40*E* − 02	3	DRD2/SLC18A2/SLC6A3
		Parkinson disease	2.60*E* − 02	3	DRD2/SLC18A2/SLC6A3

**Table 6:** The details of eight target miRNAs.

Tissue	miRNAs	mRNA targets associated with the hub genes	Reference
Substantia nigra	hsa-miR-532-5p	KCNB1	Briggs et al. [55]
	hsa-miR-23b-3p	SLC18A2	Su et al. [56]
	hsa-miR-198	EN1/KCNB1	Cardo et al. [57]
	hsa-miR-330-5p	CACNA1E/DRD2	Cardo et al. [57]
	hsa-miR-339-5p	CACNA1E	Cardo et al. [57]
	hsa-miR-485-5p	KCNB1	Cardo et al. [57]
	hsa-miR-34a-5p	NR4A2/CACNA1E	Su et al. [56]
	hsa-miR-7-5p	KCNB1/CACNA1E	Su et al. [56]

## Data Availability

This study obtained the gene expression datasets from the GEO database (https://www.ncbi.nlm.nih.gov/geo/). After a thorough review, we chose the GSE49036, GSE7621, GSE8397, and GSE42966. GSE49036 and GSE7621 were based on the GPL570 Platform (Affymetrix Human Genome U133 Plus 2.0 Array). GSE8397 was based on the GPL96 Platform (Affymetrix Human Genome U133A Array) and GPL97 Platform (Affymetrix Human Genome U133B Array), while GSE42966 was based on the GPL4133 Platform (Agilent-014850 Whole Human Genome Microarray 4x44K G4112F). The data are freely available online.

## Supplementary Materials

Supplementary Materials Supplementary table 1: 86 common DEGs between GSE49036 and GSE7621. Supplementary table 2: GO analysis of genes in four significant nodules. Supplementary table 3: KEGG pathway analysis of genes in cluster 1.
