# Supplementary material for: Identification of Hub Genes and Potential Molecular Pathogenesis in Substantia Nigra in Parkinson's Disease via Bioinformatics Analysis
Source: Parkinsons Dis. 2023 Apr 14;2023:6755569. doi: 10.1155/2023/6755569 (PMC10121343; doi:10.1155/2023/6755569)
Supplement: Supplementary Materials — Supplementary table 1: 86 common DEGs between GSE49036 and GSE7621. Supplementary table 2: GO analysis of genes in four significant nodules. Supplementary table 3: KEGG pathway analysis of genes in cluster 1. [file 6755569.f1.zip › supplementary table 1- 86 common DEGs between GSE49036 and GSE7621. (2).docx]

Common Differently Expressed Genes in GSE49036 and GSE7621

| **Gene**  **symbol** | **P-value** | | **Log2Fold change** | | **Up/Down** |
| --- | --- | --- | --- | --- | --- |
|  | **GSE49036** | **GSE7621** | **GSE49036** | **GSE7621** |  |
| *RALYL* | 1.24E-02 | 2.53E-02 | -1.10 | -1.01 | Down |
| *KLHL13* | 1.54E-03 | 8.76E-03 | -1.38 | -1.07 | Down |
| *STYK1* | 1.70E-02 | 4.14E-02 | -1.30 | -1.31 | Down |
| *PCSK1* | 2.93E-03 | 7.32E-03 | -2.07 | -1.90 | Down |
| *RAB3C* | 3.09E-02 | 2.13E-02 | -1.40 | -1.65 | Down |
| *SLC8A1* | 4.37E-04 | 2.12E-03 | -1.36 | -1.57 | Down |
| *SYNGR3* | 1.49E-02 | 3.43E-02 | -1.34 | -1.08 | Down |
| *CFAP46* | 2.79E-03 | 2.49E-02 | -1.16 | -1.44 | Down |
| *HSPA1L* | 4.94E-04 | 1.33E-03 | 1.52 | 1.46 | Up |
| *SLC2A13* | 1.92E-03 | 1.02E-02 | -1.56 | -1.21 | Down |
| *CACNB3* | 6.44E-04 | 1.98E-03 | -1.26 | -1.16 | Down |
| *CACNA1E* | 2.19E-03 | 3.14E-02 | -1.27 | -1.20 | Down |
| *SIAH3* | 4.30E-03 | 1.82E-02 | -1.52 | -1.67 | Down |
| *ISM1* | 1.40E-04 | 9.09E-03 | -1.24 | -1.50 | Down |
| *CORO2A* | 2.86E-03 | 7.03E-03 | -1.27 | -1.04 | Down |
| *LMOD3* | 7.87E-03 | 1.37E-04 | -1.11 | -1.83 | Down |
| *FOXA2* | 5.60E-04 | 1.69E-02 | -1.02 | -1.66 | Down |
| *EN1* | 2.47E-03 | 1.70E-03 | -1.59 | -1.60 | Down |
| *CLSTN2* | 3.52E-03 | 2.87E-03 | -1.64 | -2.09 | Down |
| *CATSPERG* | 6.17E-04 | 3.15E-02 | -1.07 | -1.17 | Down |
| *SMIM10L2B* | 4.90E-04 | 7.81E-04 | -1.05 | -1.06 | Down |
| *SLC10A4* | 3.23E-02 | 5.81E-04 | -1.69 | -2.55 | Down |
| *CDH8* | 1.51E-03 | 7.76E-05 | -1.54 | -1.29 | Down |
| *PLCXD2* | 8.50E-03 | 5.00E-03 | -1.62 | -1.41 | Down |
| *TPBG* | 2.16E-03 | 7.04E-04 | -1.33 | -1.50 | Down |
| *RET* | 1.10E-02 | 4.73E-03 | -1.68 | -1.96 | Down |
| *RSPO2* | 3.99E-02 | 4.08E-04 | -1.46 | -2.71 | Down |
| *PGM2L1* | 2.48E-02 | 3.00E-02 | -1.25 | -1.10 | Down |
| *OSBPL10* | 1.00E-03 | 4.14E-04 | -1.41 | -1.20 | Down |
| *KCNJ6* | 8.48E-03 | 2.28E-03 | -1.71 | -2.22 | Down |
| *RAB27B* | 2.75E-02 | 3.22E-02 | -1.12 | -1.02 | Down |
| *FST* | 6.51E-04 | 1.58E-03 | -1.87 | -2.39 | Down |
| *DRD2* | 3.67E-04 | 9.62E-04 | -1.09 | -1.14 | Down |
| *RIMBP2* | 1.90E-03 | 1.80E-02 | -1.18 | -1.12 | Down |
| *B4GALT6* | 1.13E-03 | 3.72E-02 | -1.30 | -1.20 | Down |
| *TTC39C* | 4.29E-03 | 7.94E-03 | -1.04 | -1.06 | Down |
| *EBF3* | 1.01E-02 | 2.04E-03 | -1.49 | -2.51 | Down |
| *GPRC5A* | 1.12E-03 | 6.75E-03 | -1.00 | -1.53 | Down |
| *TUB* | 3.48E-03 | 1.49E-03 | -1.00 | -1.13 | Down |
| *DIRAS2* | 8.89E-03 | 1.04E-03 | -1.26 | -1.69 | Down |
| *AGTR1* | 4.92E-03 | 1.56E-05 | -2.31 | -2.18 | Down |
| CAMK1G | 1.52E-04 | 9.48E-03 | -1.01 | -1.76 | Down |
| DOK6 | 1.31E-02 | 1.83E-02 | -1.34 | -1.55 | Down |
| KANK4 | 8.40E-04 | 1.45E-04 | -1.15 | -1.01 | Down |
| CUX2 | 1.23E-03 | 1.96E-02 | -1.73 | -1.10 | Down |
| BEND4 | 1.32E-02 | 7.14E-03 | -1.28 | -1.36 | Down |
| REEP1 | 2.57E-02 | 2.91E-02 | -1.26 | -1.03 | Down |
| GPR26 | 7.17E-03 | 5.98E-03 | -1.89 | -1.77 | Down |
| ATP2A3 | 1.89E-03 | 4.64E-03 | -1.33 | -2.13 | Down |
| FLJ30901 | 4.30E-03 | 1.27E-03 | -1.00 | -2.07 | Down |
| CBLN1 | 5.98E-03 | 9.21E-03 | -1.28 | -1.80 | Down |
| KLHL1 | 4.15E-02 | 4.86E-03 | -1.64 | -3.12 | Down |
| GBE1 | 1.18E-02 | 3.89E-04 | -1.18 | -1.27 | Down |
| SLC35D3 | 6.66E-04 | 3.92E-02 | -2.53 | -1.80 | Down |
| GPR161 | 4.99E-04 | 1.23E-03 | -1.03 | -1.57 | Down |
| SDC1 | 4.85E-03 | 6.86E-03 | -1.25 | -1.94 | Down |
| SLC18A2 | 3.26E-02 | 1.73E-05 | -1.78 | -3.15 | Down |
| KCNG3 | 4.33E-03 | 5.07E-03 | -1.02 | -1.12 | Down |
| LOC100288310 | 8.68E-03 | 4.71E-02 | -1.03 | -1.21 | Down |
| C2orf80 | 2.13E-02 | 1.72E-04 | -1.17 | -1.39 | Down |
| RDH12 | 5.17E-04 | 5.46E-03 | -1.23 | -1.32 | Down |
| CHRNB3 | 1.16E-02 | 2.05E-03 | -1.39 | -1.19 | Down |
| ALDH1A1 | 3.57E-02 | 6.36E-05 | -1.05 | -1.98 | Down |
| LOC441052 | 3.11E-03 | 8.73E-03 | -1.09 | -1.74 | Down |
| ANK1 | 1.43E-04 | 2.44E-02 | -1.42 | -1.70 | Down |
| PCDH8 | 1.01E-02 | 4.59E-04 | -1.82 | -2.20 | Down |
| CPLX2 | 7.87E-03 | 3.39E-03 | -1.15 | -1.18 | Down |
| SSTR1 | 5.11E-03 | 3.28E-04 | -1.50 | -1.36 | Down |
| CYP27C1 | 1.07E-03 | 2.17E-03 | -1.37 | -1.44 | Down |
| ROBO2 | 3.84E-03 | 7.39E-04 | -1.45 | -2.07 | Down |
| SHANK2 | 1.17E-03 | 2.79E-02 | -1.01 | -1.19 | Down |
| FGF13 | 3.32E-02 | 6.78E-03 | -1.54 | -1.68 | Down |
| CHD5 | 1.23E-03 | 4.04E-03 | -1.26 | -1.52 | Down |
| KCNB1 | 5.37E-05 | 9.35E-03 | -1.17 | -1.31 | Down |
| UNC13C | 1.21E-03 | 1.41E-04 | -1.60 | -2.57 | Down |
| GFRA1 | 8.11E-03 | 1.03E-02 | -1.12 | -1.11 | Down |
| SV2C | 1.33E-02 | 1.76E-04 | -1.94 | -2.07 | Down |
| DCC | 2.17E-03 | 7.14E-04 | -1.44 | -1.69 | Down |
| DLK1 | 7.70E-03 | 1.72E-04 | -1.99 | -2.17 | Down |
| OLFM3 | 9.57E-03 | 4.04E-02 | -1.46 | -1.16 | Down |
| DAPL1 | 3.06E-02 | 2.76E-03 | -1.08 | -2.30 | Down |
| SUSD1 | 6.87E-03 | 6.93E-03 | -1.02 | -1.54 | Down |
| CNTN6 | 2.04E-03 | 9.24E-03 | -1.39 | -1.51 | Down |
| SLC6A3 | 1.04E-02 | 1.69E-04 | -1.68 | -2.50 | Down |
| HSPA1B | 9.17E-04 | 9.84E-04 | 1.22 | 1,32 | Up |
| NR4A2 | 9.96E-04 | 1.71E-02 | -1.45 | -1.10 | Down |
