# Supplementary material for: Identification of Hub Genes and Potential Molecular Pathogenesis in Substantia Nigra in Parkinson's Disease via Bioinformatics Analysis
Source: Parkinsons Dis. 2023 Apr 14;2023:6755569. doi: 10.1155/2023/6755569 (PMC10121343; doi:10.1155/2023/6755569)
Supplement: Supplementary Materials — Supplementary table 1: 86 common DEGs between GSE49036 and GSE7621. Supplementary table 2: GO analysis of genes in four significant nodules. Supplementary table 3: KEGG pathway analysis of genes in cluster 1. [file 6755569.f1.zip › supplementary table 2- GO analysis of genes in four significant nodules. (2).docx]

Ontological Analysis of Genes in Cluster1-4

| **Category** | **GO ID** | **Term** | **P-value** | **Count** | **Genes** |
| --- | --- | --- | --- | --- | --- |
| Cluster1-BP | 0071542 | dopaminergic neuron differentiation | 1.90E-05 | 3 | *EN1/FOXA2/NR4A2* |
|  | 0042220 | response to cocaine | 5.70E-05 | 3 | *DRD2/EN1/SLC6A3* |
|  | 0001975 | response to amphetamine | 6.30E-05 | 3 | *DRD2/NR4A2/*  *SLC18A2* |
|  | 0008344 | adult locomotory behavior | 9.60E-05 | 3 | *EN1/FOXA2/NR4A2* |
|  | 0007626 | locomotory behavior | 2.90E-04 | 3 | *DRD2/SLC18A2/*  *SLC6A3* |
|  | 0045944 | positive regulation of transcription from RNA polymerase II promoter | 4.10E-03 | 4 | *DRD2/EN1/FOXA2/*  *NR4A2* |
| Cluster2-BP | 0007268 | chemical synaptic transmission | 2.50E-03 | 3 | *CACNB3/UNC13C/*  *CACNA1E* |
|  | 0007528 | neuromuscular junction development | 9.70E-03 | 2 | *CACNB3/UNC13C* |
|  | 0006816 | calcium ion transport | 2.70E-02 | 2 | *CACNB3/CACNA1E* |
|  | 0034765 | regulation of ion transmembrane transport | 4.10E-02 | 2 | *CACNB3/CACNA1E* |
|  | 0072659 | protein localization to plasma membrane | 4.80E-02 | 2 | *CACNB3/RAB3C* |
| Cluster3-BP | 0035860 | glial cell-derived neurotrophic factor receptor signaling pathway | 9.40E-04 | 2 | *GFRA1/RET* |
|  | 0007169 | transmembrane receptor protein tyrosine kinase signaling pathway | 1.40E-02 | 2 | *DOK6/ RET* |
|  | 0043410 | positive regulation of MAPK cascade | 1.70E-02 | 2 | *DOK6/ RET* |
| Cluster4-BP | 1900454 | positive regulation of long term synaptic depression | 7.30E-04 | 2 | *KCNB1/CBLN1* |
| Cluster1-CC | 0099055 | integral component of postsynaptic membrane | 1.50E-02 | 2 | *DRD2/ SLC6A3* |
|  | 0099056 | integral component of presynaptic membrane | 1.90E-02 | 2 | *DRD2/ SLC6A3* |
|  | 0030672 | synaptic vesicle membrane | 2.40E-02 | 2 | *DRD2/ SLC18A2* |
|  | 0000785 | chromatin | 3.40E-02 | 3 | *EN1/FOXA2/NR4A2* |
| Cluster2-CC | 0045202 | synapse | 2.50E-04 | 4 | *RIMBP2/CACNB3/ CACNA1E/UNC13C* |
|  | 0005886 | plasma membrane | 4.40E-03 | 6 | *RAB3C/RIMBP2/ CACNA1E/CAMK1G/*  *CATSPERG/UNC13C* |
|  | 0005891 | voltage-gated calcium channel complex | 8.60E-03 | 2 | *CACNB3/CACNA1E* |
| Cluster3-CC | 0098797 | plasma membrane protein complex | 1.60E-03 | 2 | *GFRA1/RET* |
|  | 0043235 | receptor complex | 2.10E-02 | 2 | *GFRA1/RET* |
|  | 0030424 | axon | 3.50E-02 | 2 | *GFRA1/RET* |
|  | 0043025 | neuronal cell body | 3.90E-02 | 2 | *GFRA1/RET* |
| Cluster4-CC | 0043083 | synaptic cleft | 1.90E-03 | 2 | *CDH8/ CBLN1* |
|  | 0045211 | postsynaptic membrane | 1.60E-02 | 2 | *KCNB1/CBLN1* |
|  | 0098978 | glutamatergic synapse | 3.60E-02 | 2 | *CDH8/ CBLN1* |
| Cluster1-MF | 0035240 | dopamine binding | 2.20E-03 | 2 | *DRD2/ SLC6A3* |
|  | 0008504 | monoamine transmembrane transporter activity | 2.90E-03 | 2 | *SLC18A2/ SLC6A3* |
|  | 1990837 | sequence-specific double-stranded DNA binding | 1.20E-02 | 3 | *EN1/FOXA2/NR4A2* |
| Cluster2-MF | 0008331 | high voltage-gated calcium channel activity | 2.30E-03 | 2 | *CACNB3/CACNA1E* |
|  | 0005245 | voltage-gated calcium channel activity | 9.10E-03 | 2 | *CACNB3/CACNA1E* |
|  | 0005516 | calmodulin binding | 4.30E-02 | 2 | *CAMK1G/UNC13C* |
| Cluster3-MF | 0038023 | signaling receptor activity | 2.40E-02 | 2 | *GFRA1/RET* |
