# Supplementary material for: Identification of Hub Genes and Potential Molecular Pathogenesis in Substantia Nigra in Parkinson's Disease via Bioinformatics Analysis
Source: Parkinsons Dis. 2023 Apr 14;2023:6755569. doi: 10.1155/2023/6755569 (PMC10121343; doi:10.1155/2023/6755569)
Supplement: Supplementary Materials — Supplementary table 1: 86 common DEGs between GSE49036 and GSE7621. Supplementary table 2: GO analysis of genes in four significant nodules. Supplementary table 3: KEGG pathway analysis of genes in cluster 1. [file 6755569.f1.zip › supplementary table 3- KEGG pathway analysis of genes in Cluster 1. (2).docx]

KEGG Pathways of Genes in Cluster1

| **Category** | **Term** | **P-value** | **Count** | **Genes** |
| --- | --- | --- | --- | --- |
| KEGG Pathway | Dopaminergic synapse | 4.10E-05 | 4 | *DRD2/SLC18A2/*  *KCNJ6/SLC6A3* |
|  | Cocaine addiction | 3.50E-04 | 3 | *DRD2/SLC18A2/*  *SLC6A3* |
|  | Alcoholism | 5.00E-03 | 3 | *DRD2/SLC18A2/*  *SLC6A3* |
|  | Parkinson disease | 1.00E-02 | 3 | *DRD2/SLC18A2/*  *SLC6A3* |
|  | Amphetamine addiction | 4.20E-02 | 2 | *SLC18A2/SLC6A3* |
|  | Synaptic vesicle cycle | 4.70E-02 | 2 | *SLC18A2/SLC6A3* |
